# Supplementary material for: Common and varied molecular responses of Escherichia coli to five different inhibitors of the lipopolysaccharide biosynthetic enzyme LpxC
Source: J Biol Chem. 2024 Mar 6;300(4):107143. doi: 10.1016/j.jbc.2024.107143 (PMC10998244; doi:10.1016/j.jbc.2024.107143)
Supplement: Supplemental Figures S1–S10 and Tables S1 and S2 [file mmc1.pdf]

Supplementary figures and tables

Common and varied molecular responses of *Escherichia coli* to five different inhibitors of the lipopolysaccharide biosynthetic enzyme LpxC

Anna-Maria Möller[1], Melissa Vázquez-Hernández[2], Blanka Kutscher[1], Raffael Brysch[1], Simon Brückner[1], Emily C. Marino[1], Julia Kleetz[1], Christoph HR Senges[2], Sina Schäkermann[2], Julia E Bandow[2], Franz Narberhaus[1#]

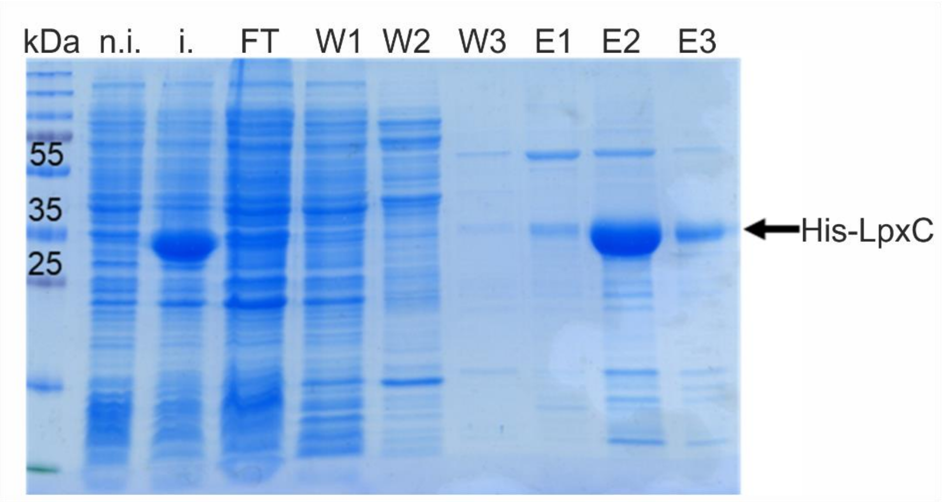

FIGURE S1 NiNTA-based purification of N-terminal His-tagged LpxC. 20 ng/ml AHT was added to exponential *E. coli* BL21 [DE3] cells harboring the plasmid pBO2382 to induce LpxC overproduction. Protein fractions were visualized by SDS-PAGE. The protein composition of 15 µl cell suspension (OD_{600}= 0.5) before or after addition of AHT (non-induced (n.i.); induced, (i.)) as well as the flow-through (FT), the wash fractions (W1-W3) and the elution fractions (E1-E3) were analyzed by Coomassie® Brilliant Blue G-250 staining. The PageRuler™ Plus Prestained Protein Ladder (Thermo Fisher Scientific) served as marker.

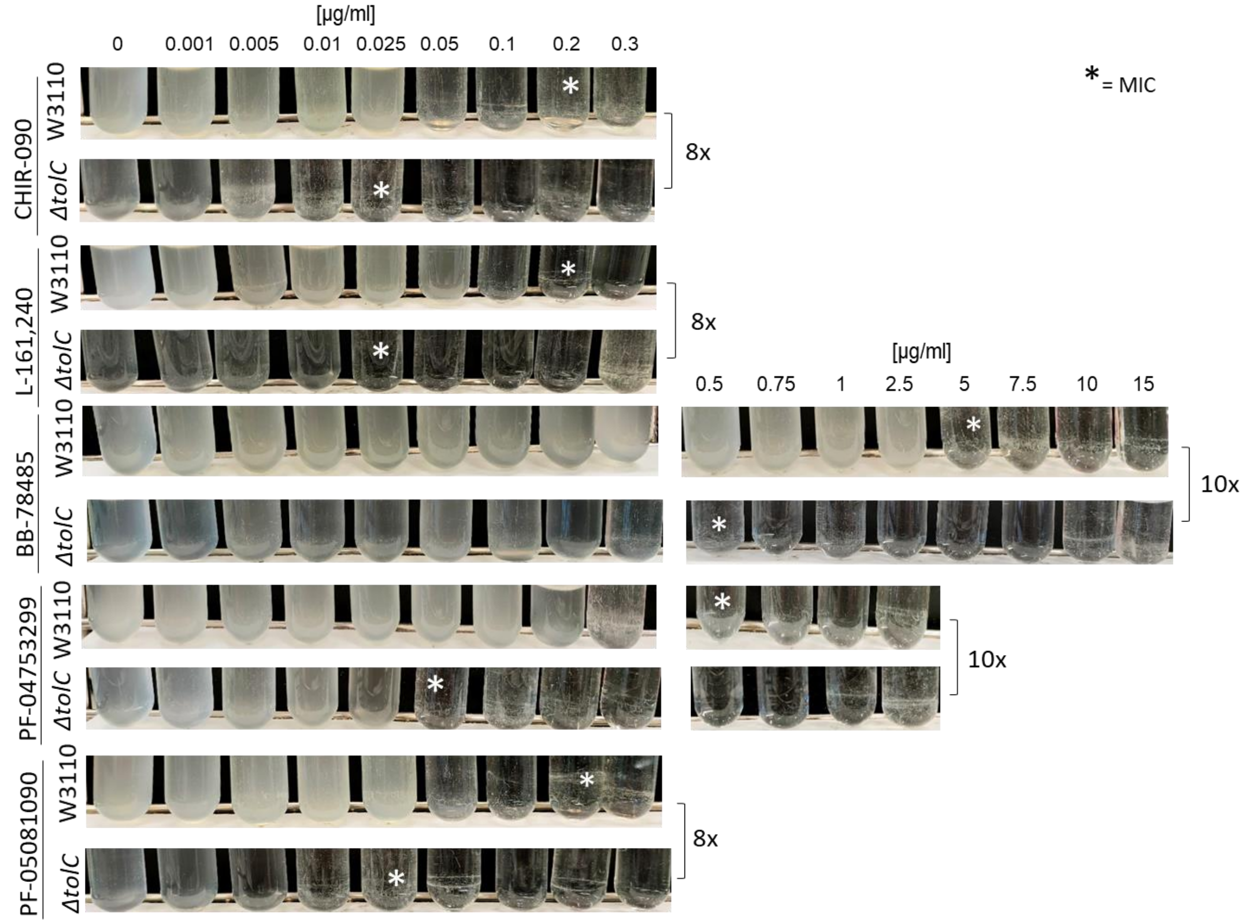

FIGURE S2 Determination of the minimal inhibitory concentration (MIC). The growth inhibiting effect of the indicated concentrations of LpxC inhibitors, solubilized in DMSO, were tested in M9 minimal medium for *E. coli* W3110 and the efflux pump-deficient *E. coli* W3110 Δ*tolC* mutant strain. An asterisk indicates the concentration that inhibited bacterial growth. The differences in sensitivity between the WT and the Δ*tolC* mutant are indicated to the right.

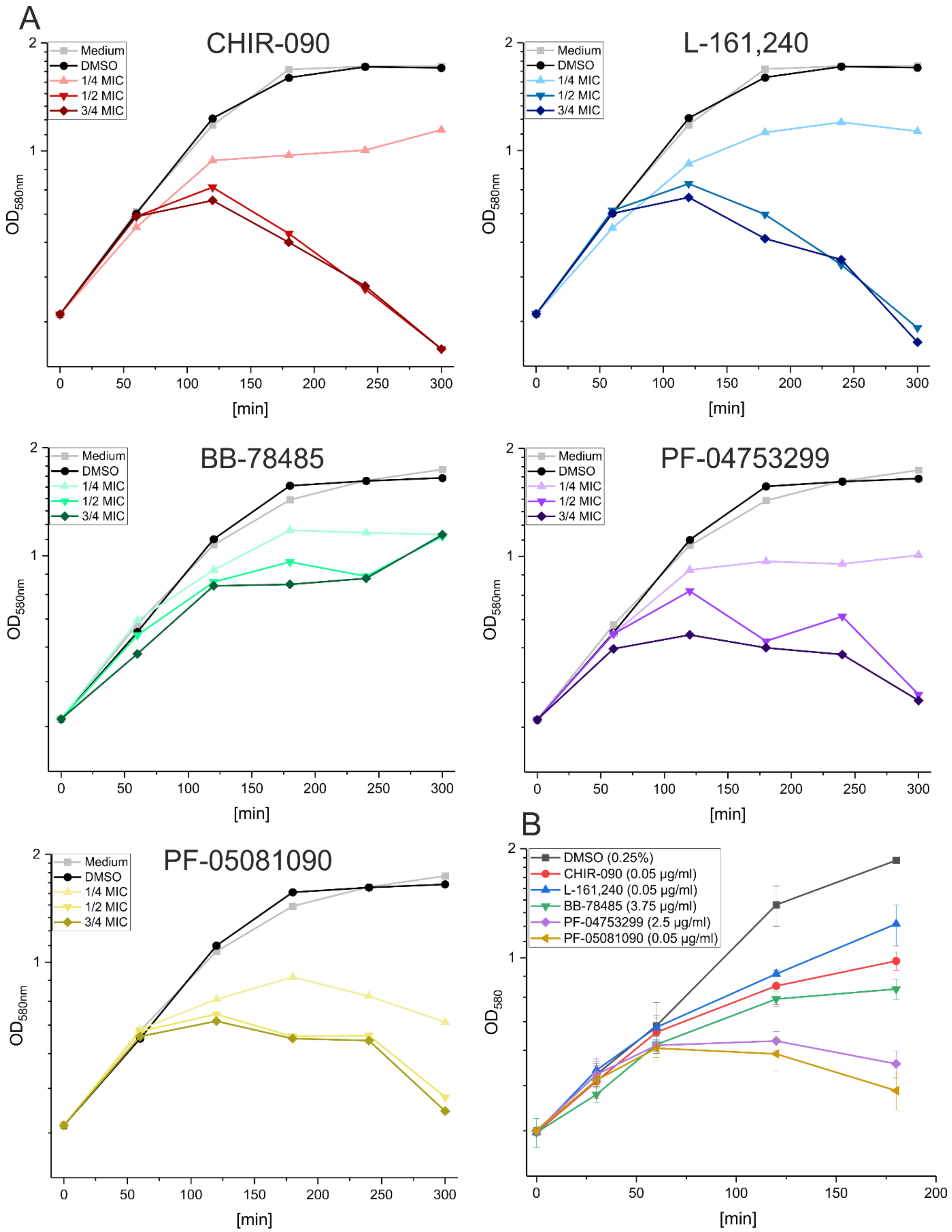

FIGURE S3 Determination of the physiologically effective concentrations (PEC). A) *E. coli* W3110 was cultivated in M9 minimal medium until exponential phase. Then, the culture was split and different LpxC inhibitor concentrations, dependent on the previously determined MIC (Table 1), were added to the subcultures. The PEC is defined as concentration needed to suppress grow for at least 30% within 120 min after exposure in comparison to the control culture, which contained DMSO. The growth was followed for five hours after compound addition. B) Growth of biological triplicates after addition of the PEC of compounds for the radioactive pulse-chase labeling experiments.

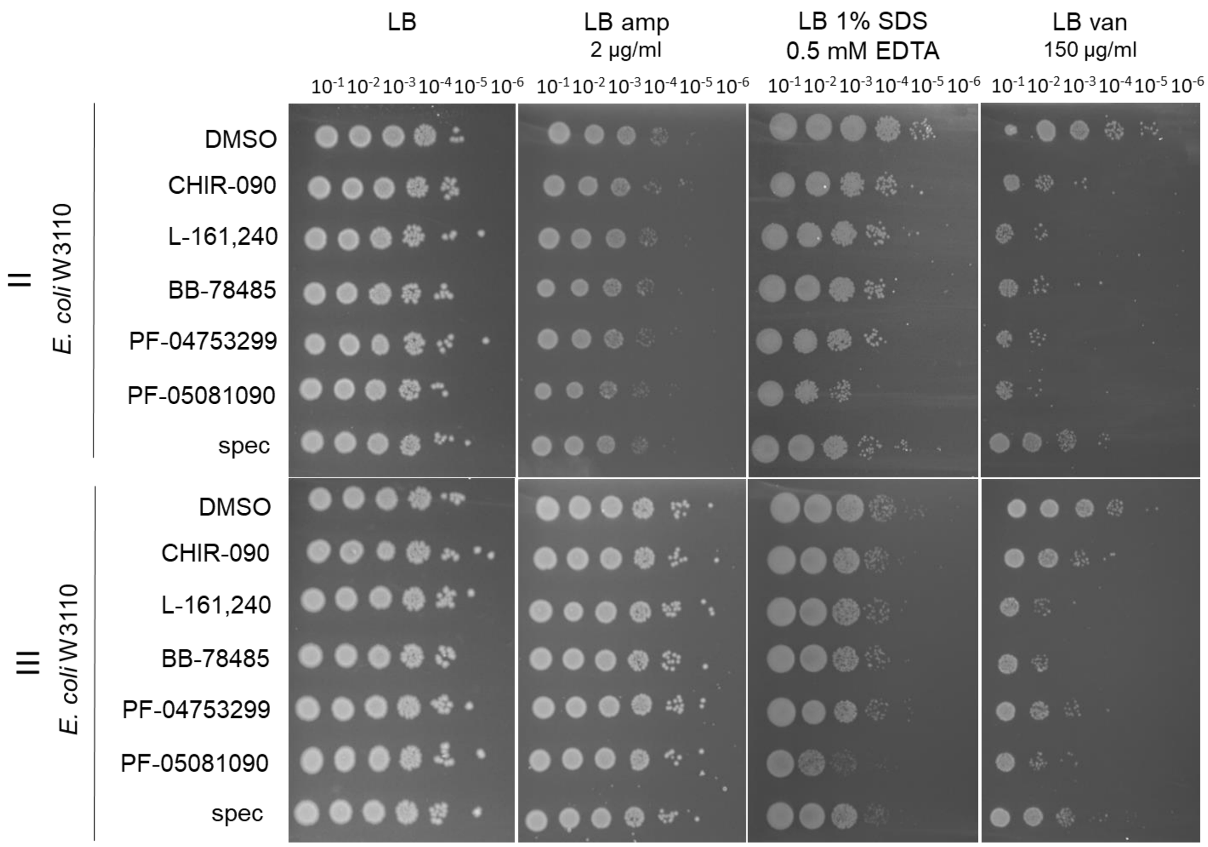

FIGURE S4 Biological replicates (II and III) of the susceptibility of LpxC inhibitor pre-treated *E. coli* W3110 to membrane targeting stressors, as shown in Figure 5.

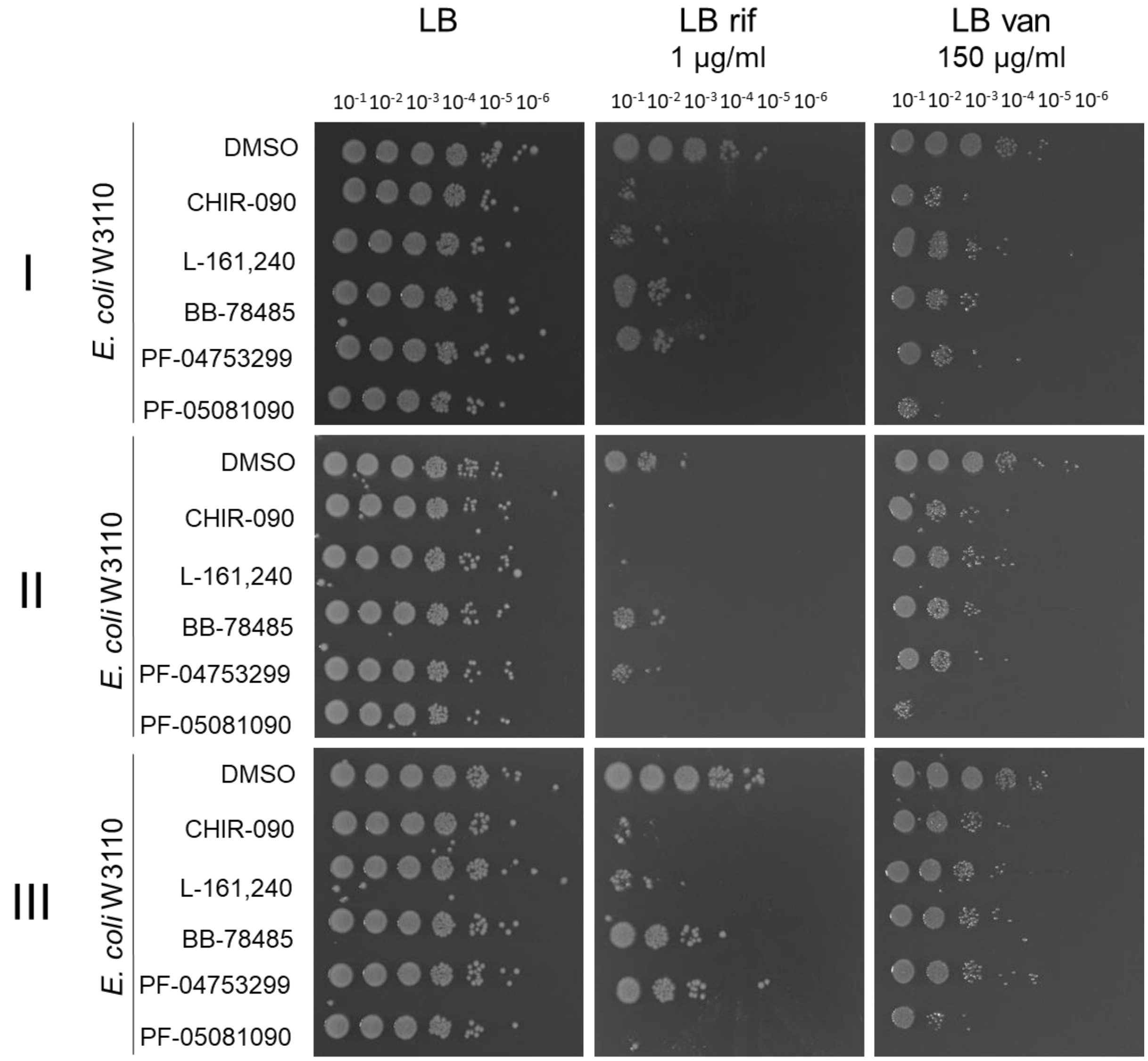

FIGURE S5 Susceptibility of *E. coli* W3110 pre-treated with LpxC inhibitors to rifampin. Washed pre-treated *E. coli* W3110 cells (exposed to ¼ MIC of the inhibitors for two hours in LB medium) were diluted from 10^{-1} to 10^{-6} in 0.9% NaCl and then spotted onto different agar plates (LB, LB rif and LB van). Pictures were taken after overnight incubation at 37°C.

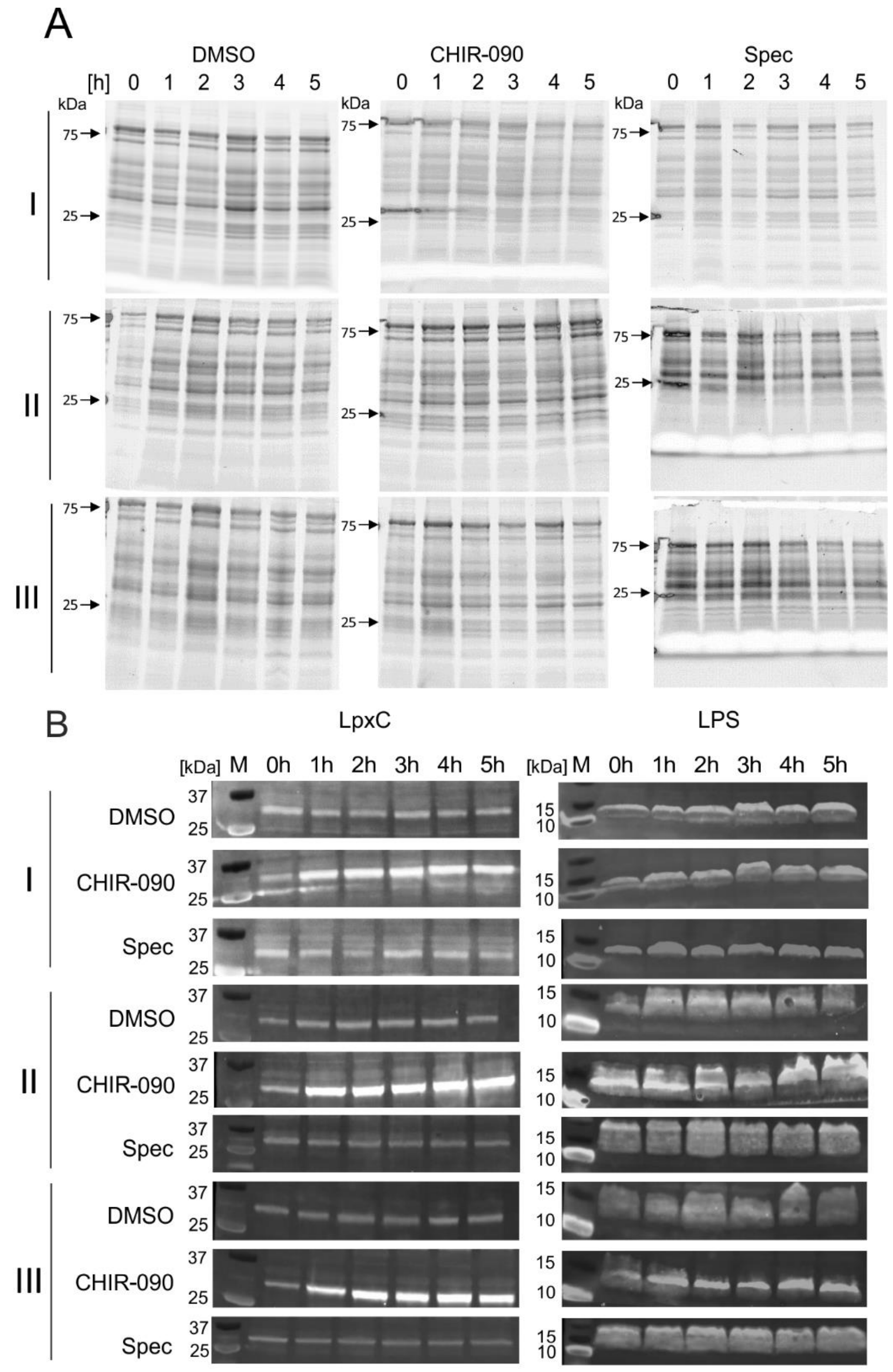

C

LpxC

	p-value		
	DMSO	CHIR-090	Spec
T=0h /T=1h	0,905	**0,000**	0,030
T=0h /T=2h	0,792	**0,007**	**0,025**
T=0h /T=3h	0,484	**0,048**	0,112
T=0h /T=4h	0,431	**0,006**	0,773
T=0h /T=5h	0,807	**0,004**	0,480

LPS

	p-value		
	DMSO	CHIR-090	Spec
T=0h /T=1h	0,258	0,899	**0,009**
T=0h /T=2h	0,656	0,419	0,830
T=0h /T=3h	0,800	0,581	0,334
T=0h /T=4h	**0,010**	**0,027**	0,277
T=0h /T=5h	0,427	0,071	0,101

FIGURE S6 SDS-PAGE and western blot analysis of the LpxC and LPS level after exposure to CHIR-090. The growth of exponential *E. coli* W3110 in M9 minimal medium after addition of DMSO, CHIR-090 (2x MIC; 0.4 µg/ml) or spectinomycin (Spec; 300 µg/ml) was tracked for 5 hours. Samples were taken every hour and directly frozen in liquid nitrogen. After harvesting, the cell pellets were

resuspended in TE-buffer and loading dye according to their optical density. A) 15 µl of each sample was subjected to SDS-PAGE. Precision Plus Prestained Western C (BioRad) was used as marker. Total protein per lane was quantified with the ImageLab (BioRad) software and was used for the normalization of the fluorescence signals. B) Via fluorescent immunodetection LpxC and LPS levels were quantified with the software ImageLab (BioRad). Whereas the LpxC signal increased after addition of CHIR-090, the LPS level remains relatively stable. In the control treatments with DMSO and Spec, all values remained at the original baseline level. The experiment was performed in triplicates (I, II, III). C) Unpaired, two-sample t-test of the quantified bands (p-value <0.05 in bold).

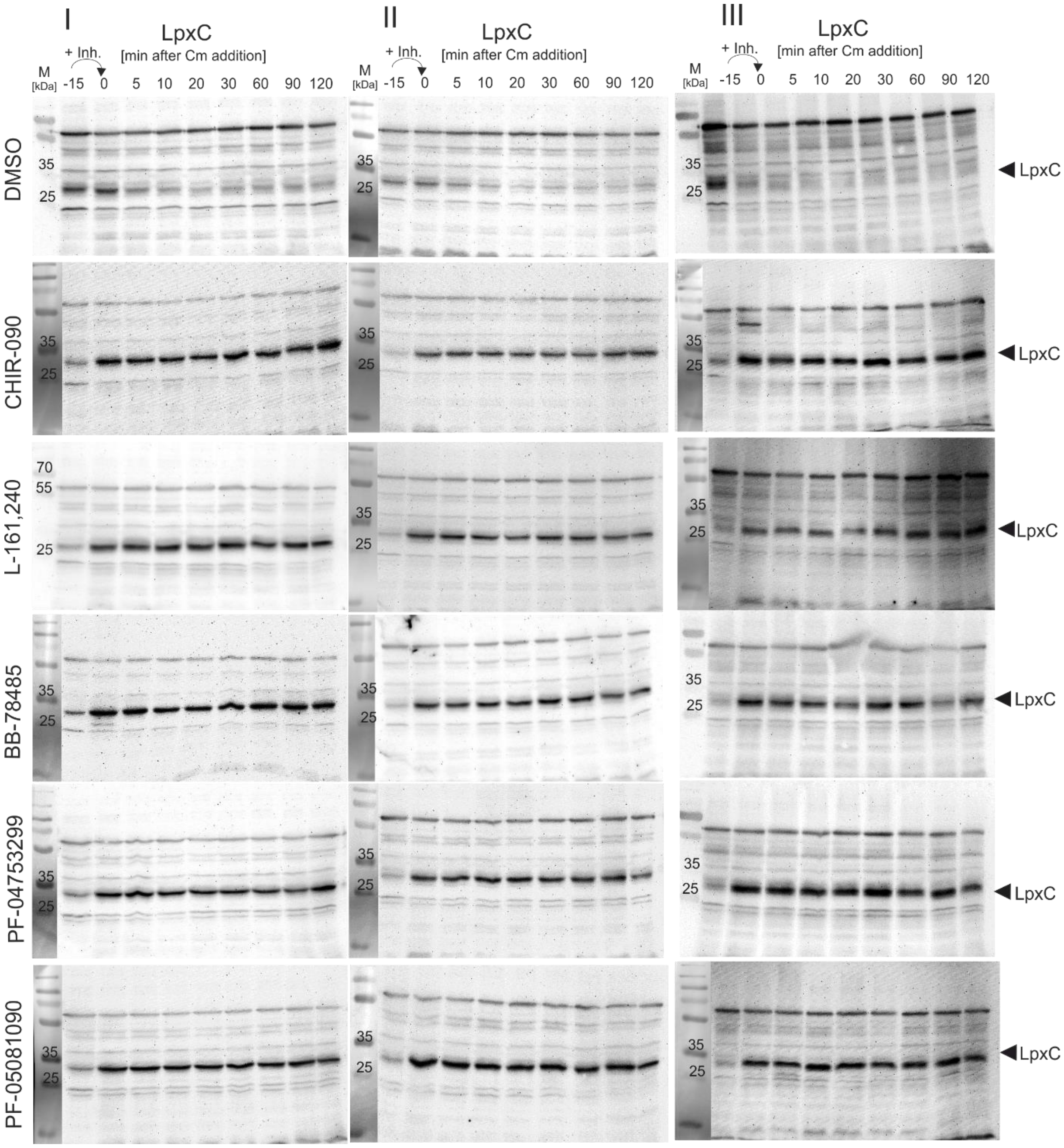

FIGURE S7 Replicates showing the increased stability of LpxC upon addition of a LpxC inhibitor. The experiment was done as described in the legend to Figure 7.

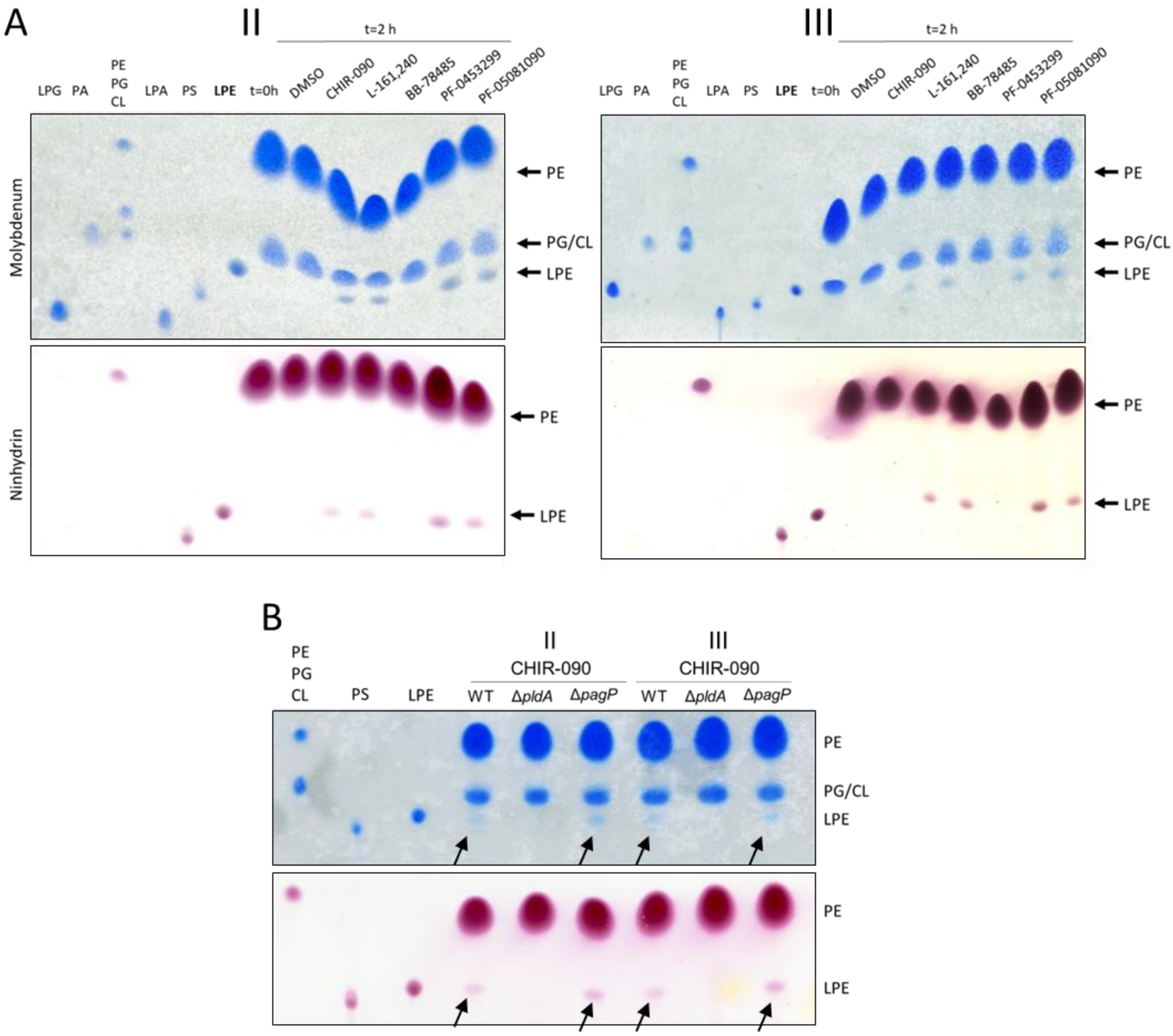

FIGURE S8 Biological replicates (II and III) of the thin layer chromatography (TLC). *E. coli* W3110 was cultivated in M9 minimal medium until exponential phase, then the culture was divided (t= 0h) and treated with DMSO or ½ MIC of the LpxC inhibitors for 2 h. Lipids from cells pellets according to OD_{580nm}=10 in 1 ml were extracted and separated via TLC using chloroform:methanol:water (65:25:4) as mobile phase. Phospholipids were visualized using molybdenum blue spray reagent and lipids with free amino groups were stained with a ninhydrin staining solution. Lipids were identified by comparison of the retention behavior of commercially available phospholipids. B) *E. coli* BW25113 (WT) and the corresponding Keio-mutants Δ*pldA* and Δ*pagP* were cultivated in M9 minimal medium till exponential phase, then the culture was divided (t= 0h) and treated with ½ MIC of CHIR-090 for 2 h. Lipid extraction and TLC analysis was performed as described in A). LPG: lyso-phosphatidylglycerol; PA: phosphatidic acid; PE: phosphatidylethanolamine; PG: phosphatidylglycerol; CL: cardiolipin; LPA: lyso-phosphatidic acid; PS: phosphatidylserine; LPE: lyso-phosphatidylethanolamine.

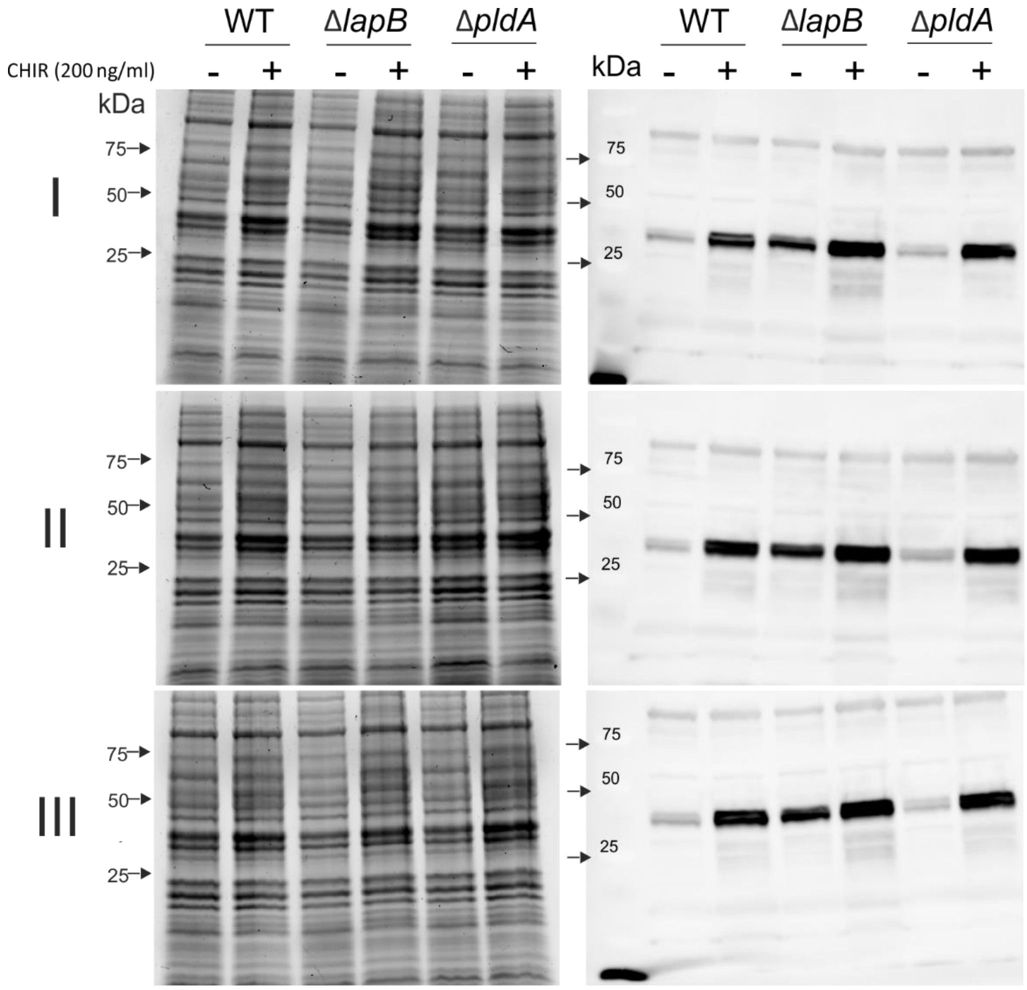

FIGURE S9 Steady state level of LpxC in *E. coli* BW25113 strains in the absence or presence of CHIR-090. Samples were equalized by adjusting their optical density. After SDS-PAGE separation, proteins were blotted onto nitrocellulose. The experiment was performed in triplicates and the overall protein content of each sample is visualized via the stain-free channel (left). LpxC was detetcted via chemiluminescence with a polyclonal LpxC antiserum and an HRP-coupled goat anti-rabbit antibody (right).

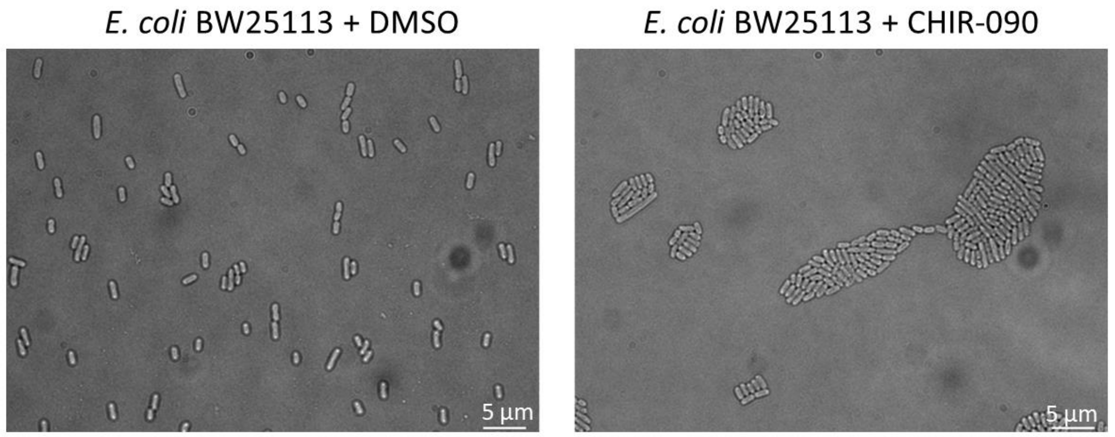

FIGURE S10 *E. coli* BW25113 cells tend to auto-aggregate in LB medium in response to exposure to CHIR-090. Microscopy was performed 2 h after treatment with 200 ng/ml CHIR-090. Scale bar = 5 µm

Table S1 Regulated proteins of *E. coli* W3110 after exposure to the five LpxC inhibitors, CHIR-090, L-161,240, BB-78485, PF-04753299 or PF-05081090. Since the 2D-PAGE analysis was split into two runs, once analyzing the response to CHIR-090, L-161,240 and BB-78485 and in another run analyzing the response to PF-04753299 and PF-05081090, not all spot intensities could be compared among the inhibitors, indicated by grey fields. Colored fields indicate that this protein was upregulated (defined as minimum 1.6x fold signal increase) in each triplicate (I, II and III) of the response to the inhibitor.

	CHIR-090			L-161,240			BB-78485			PF-04753299			PF-05081090			
	I	II	III	I	II	III	I	II	III	I	II	III	I	II	III	Protein function
**Carbon metabolism**																
**AlkH**	1.2	1.1	1.4	1.4	1.5	0.9	1.9	2.0	1.8							KHG/KDPG aldolase
**G3P1**	1.3	1.4	1.6	1.3	1.5	1.4	2.0	2.1	1.9							Glyceraldehyde 3-phosphate dehydrogenase A
**Gal1**										2.8	1.4	1.5	2.0	2.6	2.0	Galactokinase
**GcsT**	1.2	1.2	1.1	1.2	0.9	0.9	3.4	5.9	4.9							Aminomethyltransferase
**GhrB**	1.3	2.0	1.8	2.1	2.2	1.7	2.6	2.4	3.3							Glyoxylate/hydroxypyruvate reductase B
**GpmA**	1.5	2.1	2.0	1.3	3.0	1.3	3.8	6.4	5.1							2,3-bisphosphoglycerate-dependent phosphoglycerate mutase
**Mao1**										1.6	1.7	1.7	1.6	1.5	1.5	NAD-dependent malic enzyme
**Odp1**										2.3	1.7	1.9	1.9	0.9	2.8	Pyruvate dehydrogenase E1 component
**Odp1**										1.9	1.7	3.5	2.0	1.0	5.4	Pyruvate dehydrogenase E1 component
**Odp2**										2.2	1.8	1.9	2.5	1.0	1.8	Dihydrolipoyllysine-residue acetyltransferase component of pyruvate dehydrogenase complex
**RpiA**	1.5	1.5	1.5	1.3	1.7	1.2	1.9	2.0	1.9							Ribose-5-phosphate isomerase A
**SucC**	0.9	0.5	1.8	0.3	0.2	0.3	2.2	3.6	6.4							Succinate-CoA ligase [ADP-forming] subunit beta
**DNA synthesis**																
**CysQ**	2.6	2.4	1.7	1.3	13.5	1.5	2.4	4.6	3.5							3'(2')5'-bisphosphate nucleotidase
**Dcd**	1.1	1.1	1.7	1.3	1.1	0.9	2.4	2.1	2.4							dCTP deaminase
**PurC**	1.5	1.3	1.8	1.2	1.5	1.1	3.9	2.4	3.7	3.7	1.7	2.5	3.7	2.3	1.6	Phosphoribosylaminoimidazole-succinocarboxamide synthase
**PyrB**	1.1	1.1	1.3	1.3	1.0	0.7	2.0	2.1	2.3							Aspartate carbamoyltransferase catalytic subunit
**PyrF**	1.5	1.7	1.7	1.2	2.0	1.2	2.2	2.1	2.0							Orotidine 5'-phosphate decarboxylase
**PyrG**	0.9	1.0	0.6	1.3	0.5	1.5	5.2	3.6	3.2							CTP synthase
**TysY**	1.2	1.8	2.0	1.4	1.2	1.9	2.2	2.0	2.2	2.6	1.4	0.7	2.0	1.8	4.1	Thymidylate synthase
**Udp**	1.2	1.4	1.7	1.3	1.4	1.2	2.7	2.8	3.4							Uridine phosphorylase
**Lipid metabolsim**																
**FabA**	2.7	2.6	3.9	1.9	3.7	2.5	2.1	1.5	2.3	2.1	1.8	2.1	3.1	2.2	1.4	3-hydroxydecanoyl-[acyl-carrier-protein] dehydratase

**FabA**	2.0	2.0	3.0	1.3	1.9	2.0	2.5	2.4	3.7	1.4	1.7	1.9	2.3	1.6	1.3	3-hydroxydecanoyl-[acyl-carrier-protein] dehydratase
**FabB**	2.3	1.8	2.6	1.2	2.2	1.4	1.6	1.8	1.9							3-oxoacyl-[acyl-carrier-protein] synthase 1
**FabB**	1.1	1.3	0.7	1.1	1.0	0.9	1.9	1.9	2.3	3.5	3.9	1.2	4.4	3.6	0.8	3-oxoacyl-[acyl-carrier-protein] synthase 1
**HdhA**	1.4	1.1	2.4	0.9	0.8	1.6	2.3	2.8	5.7							7-alpha-hydroxysteroid dehydrogenase
**KdsC**	1.2	1.3	2.5	1.3	1.5	1.5	1.9	2.5	3.7							3-deoxy-D-manno-octulosonate 8-phosphate phosphatase
**LpxC**	2.4	2.2	4.0	1.9	2.0	1.6	5.0	4.0	5.0	4.3	4.4	1.8	3.1	4.8	0.5	UDP-3-O-acyl-N-acetylglucosamine deacetylase
**WecB**	0.9	0.5	1.4	1.2	0.4	1.0	2.4	2.5	3.8							UDP-N-acetylglucosamine 2-epimerase
**protein synthesis**																
**CysJ**										1.9	1.7	2.5	2.9	1.0	2.5	Sulfite reductase [NADPH] flavoprotein alpha-component
**DapA**	0.6	0.7	1.7	0.6	0.4	1.9	2.3	2.1	3.0							4-hydroxy-tetrahydrodipicolinate synthase
**EfpL**	2.1	1.6	3.0	1.3	1.5	2.1	2.8	2.4	3.2							Elongation factor P-like protein
**EfTu1**	1.4	1.9	1.1	2.4	1.9	1.2	3.6	2.4	2.4							Elongation factor Tu 1
**Fur**	1.6	1.6	1.7	1.2	1.9	1.0	3.6	3.6	4.6							Ferric uptake regulation protein
**His6**	1.4	1.2	1.6	1.1	1.4	1.3	2.2	2.7	2.9							Imidazole glycerol phosphate synthase subunit HisF
**HisJ**	1.6	2.2	3.3	2.1	2.5	2.6	1.0	1.1	1.7	2.9	1.6	0.8	2.4	1.8	0.6	Histidine-binding periplasmic protein
**IlvD**	0.7	0.4	0.1	1.1	0.3	1.2	4.7	1.9	2.4							Dihydroxy-acid dehydratase
**LeuD**	1.3	1.4	1.9	1.2	1.7	1.5	2.8	3.7	4.4							3-isopropylmalate dehydratase small subunit
**MetB**	0.7	0.6	0.8	0.8	0.5	0.9	5.0	2.2	3.1							Cystathionine gamma-synthase
**MtnN**										2.9	1.6	0.8	2.4	1.8	0.6	5'-methylthioadenosine/S-adenosylhomocysteine nucleosidase
**Sye**	0.8	1.0	0.4	1.3	0.8	0.8	3.3	1.8	2.2							Glutamate-tRNA ligase
**Syl**										2.6	1.7	1.6	2.3	1.0	6.1	Leucine-tRNA ligase
**TcyJ**	1.7	1.9	3.6	2.3	2.3	3.2	2.2	1.0	2.2							L-cystine-binding protein TcyJ
**TyrB**										1.6	1.8	3.5	1.2	0.9	1.6	Aromatic-amino-acid aminotransferase
**YebC**	5.7	1.8	1.8	18.1	2.7	1.6	16.6	2.8	0.9							Probable transcriptional regulatory protein YebC
**Stress response**																
**AhpC**	2.2	2.5	2.0	1.4	2.0	1.2	1.4	1.2	2.4							Alkyl hydroperoxide reductase C
**CpdB**										3.1	2.1	14.1	2.3	1.2	1.4	2'3'-cyclic-nucleotide 2'-phosphodiesterase/3'-nucleotidase
**Dcp**										2.3	1.7	11.3	2.4	1.1	14.6	Dipeptidyl carboxypeptidase
**DksA**	2.5	2.9	2.3	0.7	1.6	1.1	1.1	1.4	1.7							RNA polymerase-binding transcription factor
**DsbA**	1.6	1.8	2.8	2.7	1.8	2.7	2.5	1.8	3.4	2.2	1.0	1.1	1.8	1.5	0.8	Thiol:disulfide interchange protein

**Hns**	1.7	1.4	1.6	1.3	1.3	1.0	2.2	2.5	4.7							DNA-binding protein H-NS
**NadE**	0.9	1.1	1.1	1.0	0.9	0.8	2.2	2.1	2.3							NH(3)-dependent NAD(+) synthetase
**LuxS**	1.2	1.7	2.5	1.1	2.4	2.0	2.4	2.8	4.8							S-ribosylhomocysteine lyase
**LuxS**	1.0	1.7	1.3	1.3	2.3	1.1	1.9	2.9	2.3							S-ribosylhomocysteine lyase
**Pnp**										2.7	1.6	2.5	3.1	0.9	1.9	Polyribonucleotide nucleotidyltransferase
**SodF**	1.5	1.3	1.6	1.4	1.1	1.1	1.9	2.4	3.0							Superoxide dismutase [Fe]
**SspA**	1.2	1.4	1.4	1.5	1.7	1.1	2.8	2.4	2.7							Stringent starvation protein A
**TatD**										3.7	1.7	2.5	3.7	2.3	1.6	3'-5' ssDNA/RNA exonuclease
**Tig**	1.9	1.6	2.6	1.2	1.5	2.0	2.3	2.3	3.2							Trigger factor
**UspA**	0.8	1.4	1.5	0.8	1.2	1.7	2.0	4.3	6.4							Universal stress protein A
**YfgM**	1.3	2.1	1.3	2.1	3.1	0.7	2.7	3.0	2.0							Ancillary SecYEG translocon subunit
**Other**																
**ElbB**	1.1	1.0	1.3	1.1	1.4	0.9	2.4	2.0	2.6							Glyoxalase
**EntA**										5.5	1.3	3.0	4.8	1.9	2.0	2,3-dihydro 2,3-dihydroxybenzoate dehydrogenase
**MoaB**	1.1	1.5	1.7	1.2	1.5	1.0	3.3	2.9	4.4							Molybdenum cofactor biosynthesis protein B
**OmpA**	1.6	1.8	2.1	0.7	1.3	1.5	1.5	2.8	1.8							Outer membrane protein A
**OpgG**										2.6	1.8	2.0	2.1	0.9	2.8	Glucan biosynthesis protein G
**uncharacterized**																
**YajQ**	1.5	1.7	2.0	1.1	1.7	1.2	2.0	2.0	2.5							unknown function
**YeeZ**	1.0	1.0	1.4	1.1	0.8	1.0	1.9	1.8	2.5							unknown function
**YfbU**	1.2	1.5	1.7	0.8	1.6	1.2	2.1	2.4	2.9							unknown function
**YhfA**	1.7	1.9	2.5	2.0	2.4	1.1	3.2	2.6	5.1							unknown function

TABLE S2 Liquid chromatography gradient

**Time [min]**	**% ACN + 0.1% FA**
0.00	1
0.50	1
4.75	40
5.00	85
5.50	85
5.60	1
7.00	1
